# Supplementary figures and images for: Ubiquitin Pathway Is Associated with Worsening Left Ventricle Function after Mitral Valve Repair: A Global Gene Expression Study
Source: Int J Mol Sci. 2020 Jul 18;21(14):5073. doi: 10.3390/ijms21145073 (PMC7404186; doi:10.3390/ijms21145073)

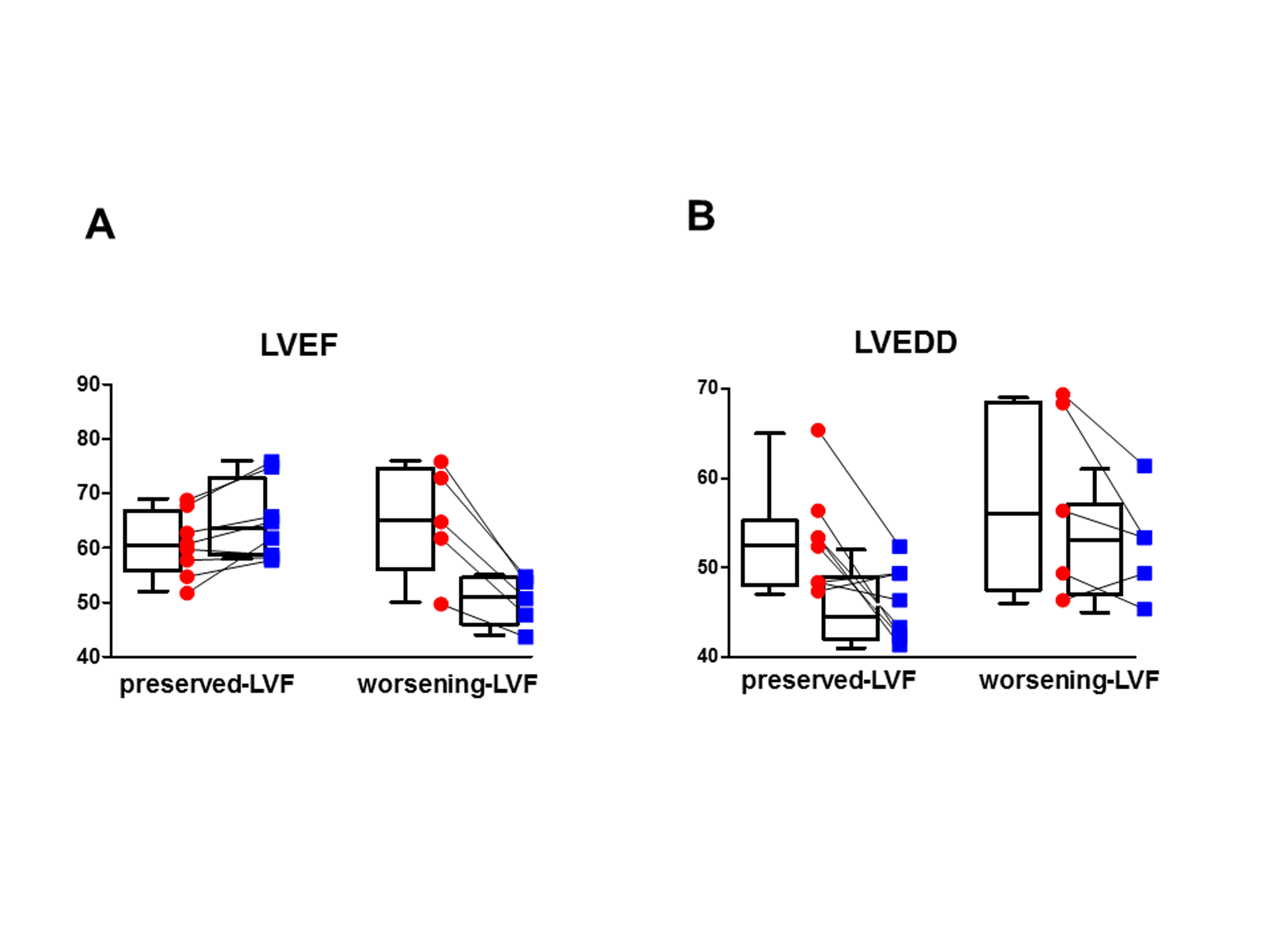

Supplement: Supplementary file 1 [file ijms-21-05073-s001.zip › Supplemental Figure 1.tif]

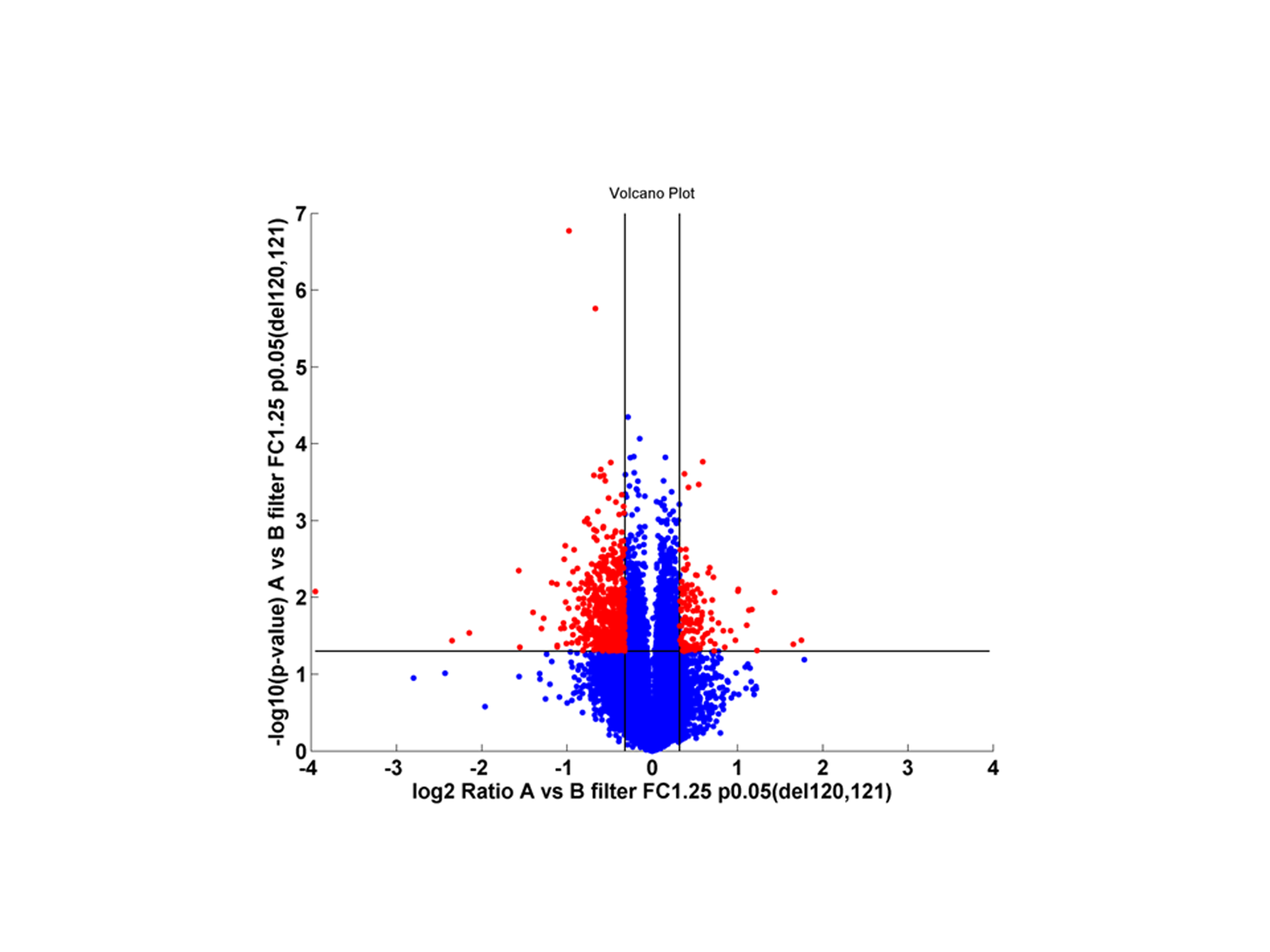

Supplement: Supplementary file 1 [file ijms-21-05073-s001.zip › Supplemental Figure 2.tif]

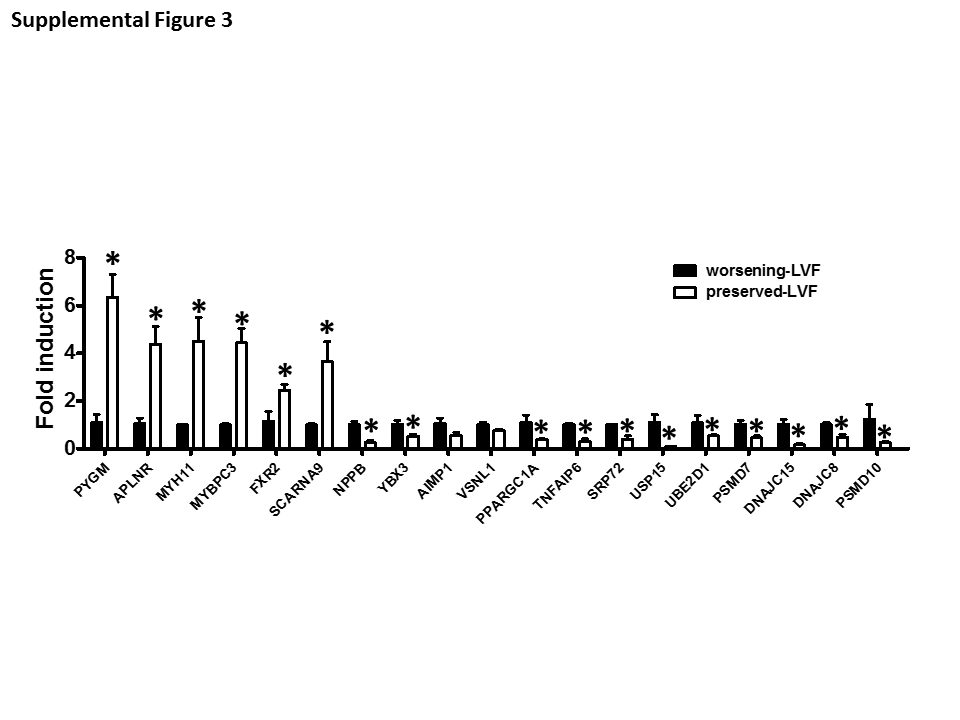

Supplement: Supplementary file 1 [file ijms-21-05073-s001.zip › Supplemental Figure 3.tif]

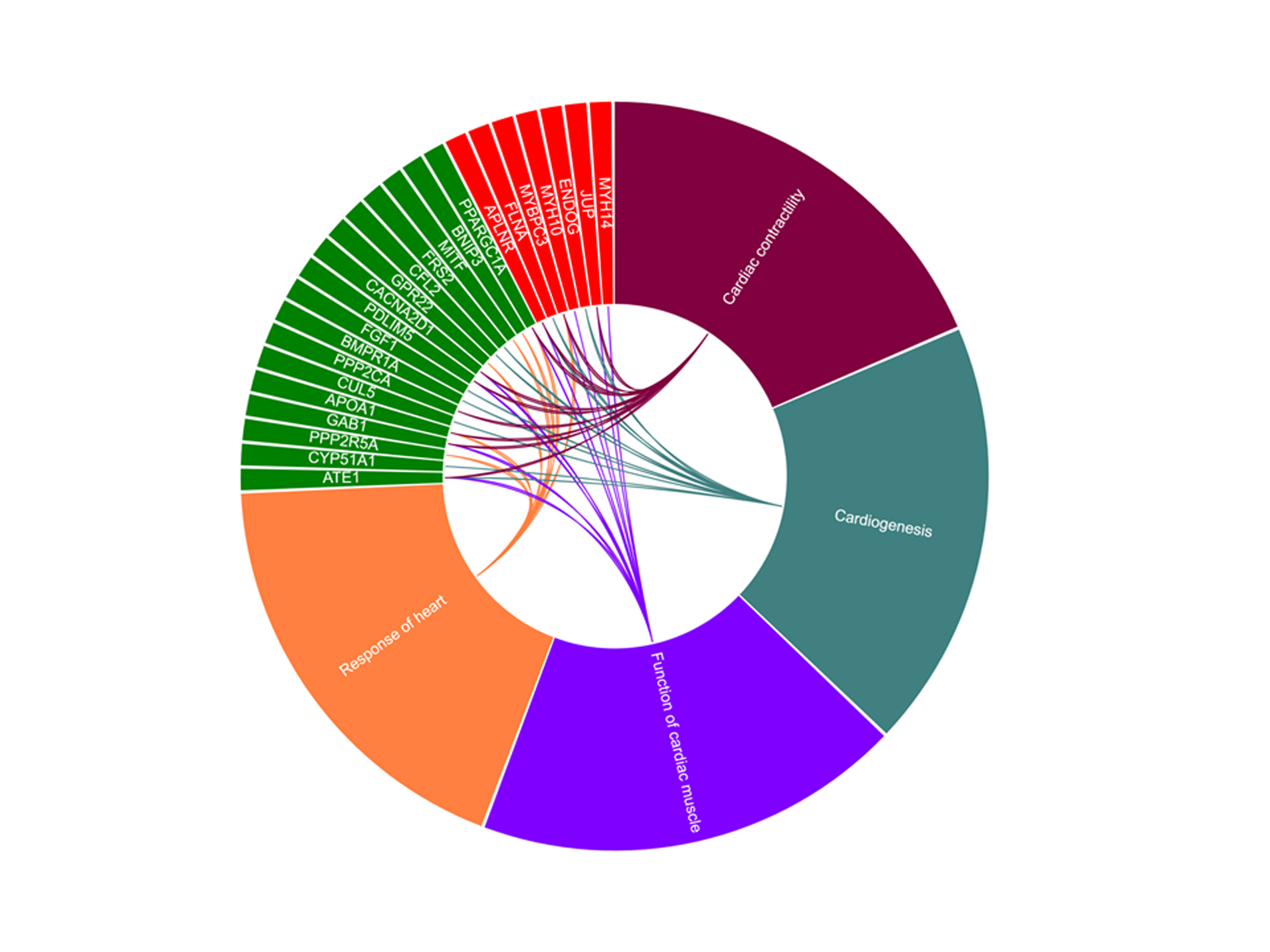

Supplement: Supplementary file 1 [file ijms-21-05073-s001.zip › Supplemental Figure 4.tif]

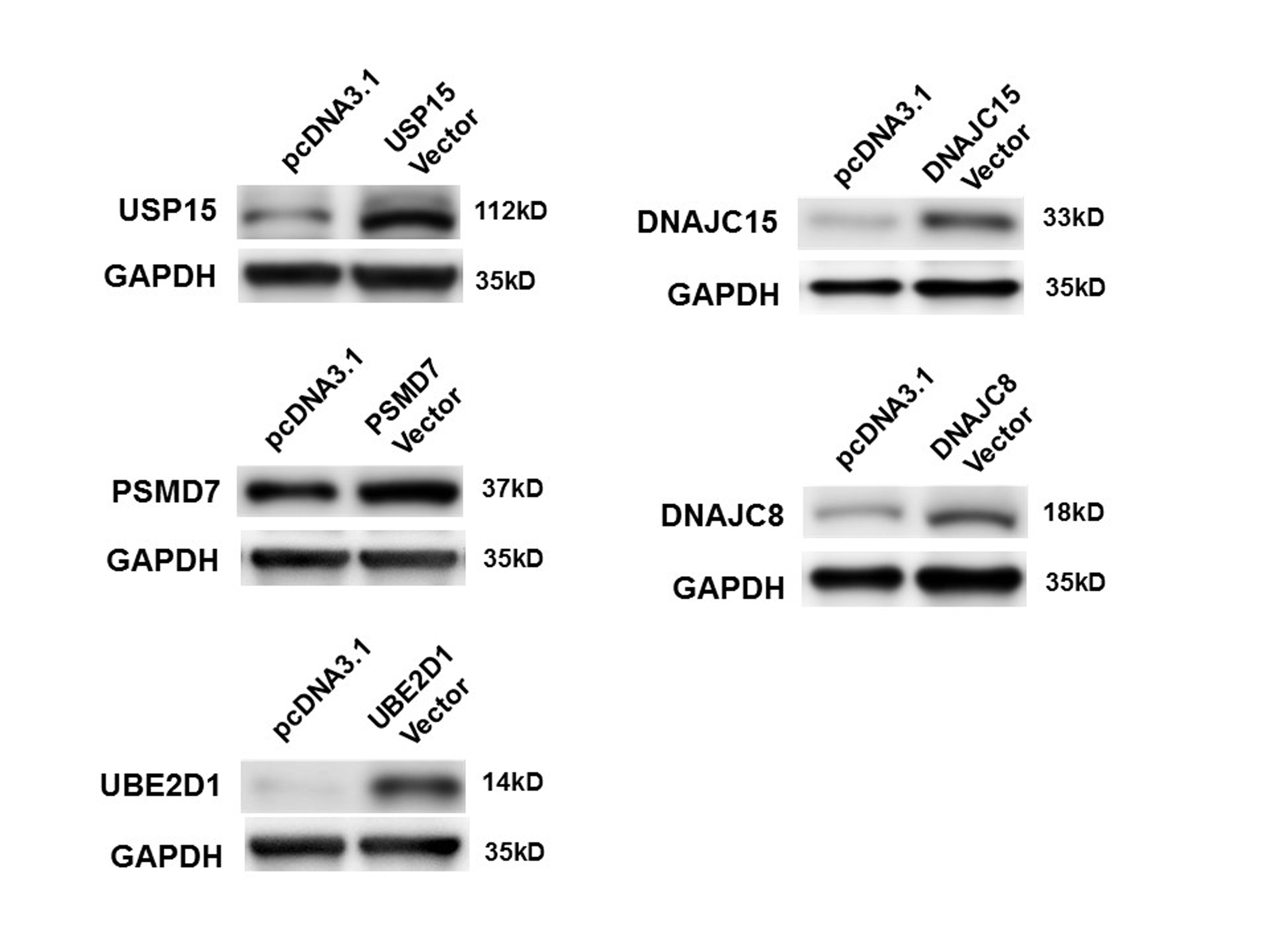

Supplement: Supplementary file 1 [file ijms-21-05073-s001.zip › Supplemental Figure 5 R1.tif]

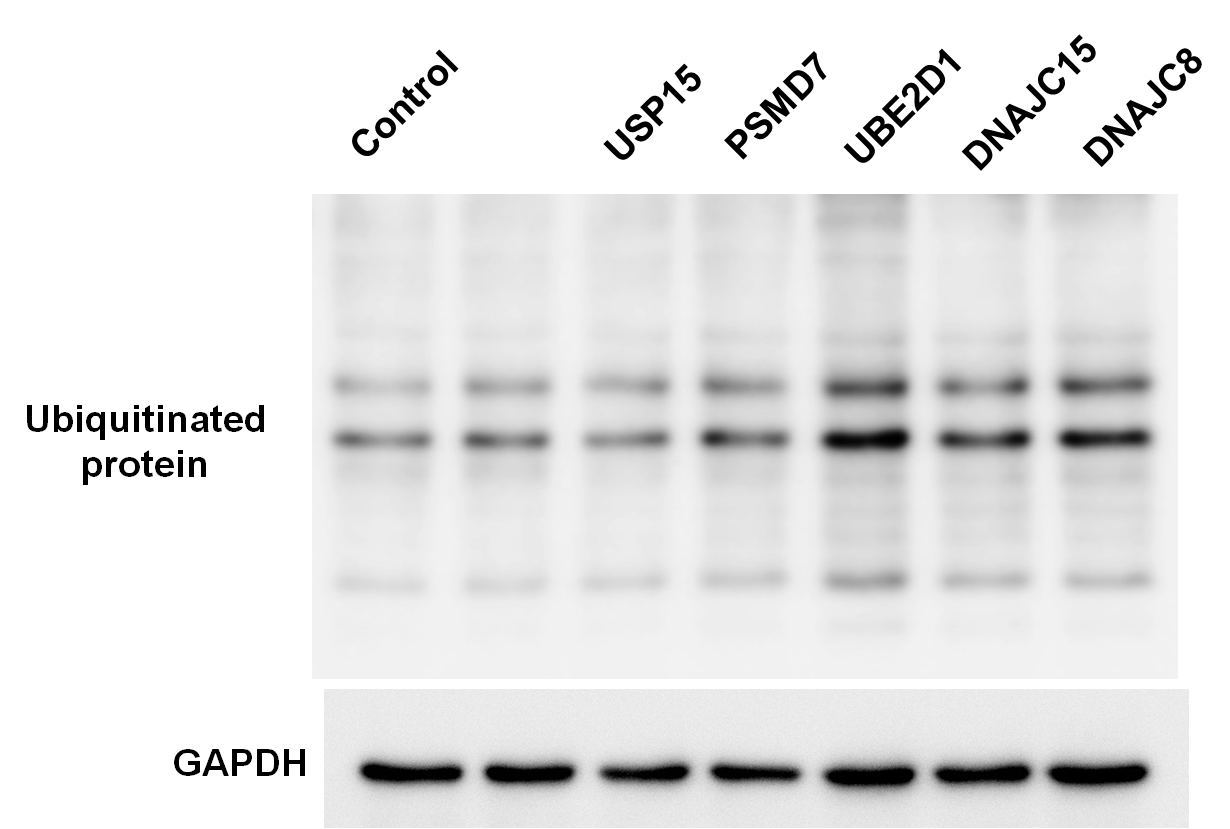

Supplement: Supplementary file 1 [file ijms-21-05073-s001.zip › Supplemental Figure 6.tif]
